# Transcriptome analysis of nitric oxide-responsive genes in upland cotton (*Gossypium hirsutum*)

**DOI:** 10.1371/journal.pone.0192367

**Published:** 2018-03-07

**Authors:** Juan Huang, Hengling Wei, Libei Li, Shuxun Yu

**Affiliations:** 1 Research Center of Buckwheat Industry Technology, Guizhou Normal University, Guiyang, Guizhou, P. R. China; 2 State Key Laboratory of Cotton Biology, Institute of Cotton Research, Chinese Academy of Agricultural Sciences, Anyang, Henan, P. R. China; National Taiwan University, TAIWAN

## Abstract

Nitric oxide (NO) is an important signaling molecule with diverse physiological functions in plants. It is therefore important to characterize the downstream genes and signal transduction networks modulated by NO. Here, we identified 1,932 differentially expressed genes (DEGs) responding to NO in upland cotton using high throughput tag sequencing. The results of quantitative real-time polymerase chain reaction (qRT-PCR) analysis of 25 DEGs showed good consistency. Gene Ontology (GO) and KEGG pathway were analyzed to gain a better understanding of these DEGs. We identified 157 DEGs belonging to 36 transcription factor (TF) families and 72 DEGs related to eight plant hormones, among which several TF families and hormones were involved in stress responses. Hydrogen peroxide and malondialdehyde (MDA) contents were increased, as well related genes after treatment with sodium nitroprusside (SNP) (an NO donor), suggesting a role for NO in the plant stress response. Finally, we compared of the current and previous data indicating a massive number of NO-responsive genes at the large-scale transcriptome level. This study evaluated the landscape of NO-responsive genes in cotton and identified the involvement of NO in the stress response. Some of the identified DEGs represent good candidates for further functional analysis in cotton.

## Introduction

Nitric oxide (NO) is a small gaseous molecule with a free radical nature. It participates in a wide range of important biological processes [1]. Since the significant role of NO in plants was realized in the mid-1900s [2], the biosynthesis of NO in plants has been extensively studied, and two NO biosynthetic pathways of plants have been described. The main pathway is dependent on nitrate reductase, which catalyzes the reduction of nitrite to NO [3, 4]. The second pathway is an arginine-dependent reaction catalyzed by NOA1 (previously referred to as NOS1) [5]. However, NOA1 was subsequently found to play a primary role in chloroplast function, and its effects on NO accumulation are likely indirect [6]. Therefore, nitrate reductase appears to be the primary enzyme for the biosynthesis of NO in plants.

NO acts as a key signaling molecule in many biological processes, including seed dormancy and germination, root organogenesis, the transition to flowering, the regulation of pollen tubes, fruit ripening, plant senescence, and responses to biotic and abiotic stresses [4, 7–10]. In addition, NO mediates the biological effects of small molecules, such as reactive oxygen species and iron [11, 12]. The NO-donor sodium nitroprusside (SNP) induces ferritin at both the mRNA and protein levels [11]. After iron treatment, NO quickly accumulates in plastids, and the expression of *AtFer1* increases [12]. NO also interacts with signal pathways involving hormones and Ca^2+^ [13–15].

NO play a role in plant responses to various abiotic stresses, such as drought, heat, and salt [8, 16]. The application of two NO donors, SNP and diethylenetriamine NO adduct (DETA/NO), leads to a significant reversal in the dry weight and water loss in wheat seedling roots [8]. Treatment of *Arabidopsis* seedlings with heat results in a reduction in endogenous NO levels, and NO functions in signaling and acts upstream of AtCaM3 in thermo tolerance [16]. In addition, NO interacts with endogenous signals, such as abscisic acid (ABA), ethylene (ET), and auxin (AUX), to regulate diverse biological processes [13, 15].

Efforts have been made to identify NO-responsive genes in plants using large-scale transcriptomic methods. In *Arabidopsis thaliana*, four transcriptomic analyses identified large amounts of differentially expressed genes (DEGs) and common transcription factor-binding sites (TFBSs) in the promoter regions of DEGs. These DEGs are related to signal transduction, hormone biosynthesis and signaling, transcription factors (TFs), disease resistance, stress responses, photosynthesis, cellular transport, and basic metabolism [17–20]. NO-responsive genes have also been identified in other plants, such as *Nicotiana tabacum* [21], *Medicago truncatula* [22], *Litchi chinensis* [23], *Betula platyphylla* [24], and *Larix olgensis* [25]. These analyses provide useful information about how NO influences downstream genes and networks under cell death, pathogenic and symbiotic conditions, in addition to triterpene biosynthesis. Upland cotton (*Gossypium hirsutum*) is an economically import species, as it produces fiber that keeps humans warm. A proteomics analysis was previously performed to identify NO-responsive proteins in cotton [26], but the NO-responsive genes in this species remain unknown. In the present study, we used a high-throughput tag-sequencing method to identify DEGs responding to NO after treatment of cotton with SNP. We obtained sets of up-regulated and down-regulated genes. Several NO-responsive genes were identified and are discussed in association with the diverse functions of NO in plants.

## Materials and methods

### Plant growth and treatment with NO donor

Seeds of *G*. *hirsutum* ecotype CCRI10 were sown in nutritional soil in a greenhouse. The daily photoperiod was set to 16 h/8 h (light/dark), and the corresponding temperatures were 30/22°C (light/dark). As NO is gaseous, we selected a widely accepted NO donor, SNP, for NO treatment [18, 23, 25]. Twenty days after germination, plants with robust and consistent growth were irrigated with 100 μM SNP or 250 μM SNP under continuous light. The control plants were treated with double-distilled water. Cotyledons were harvested at 0, 1, 3, 6, 9, 12, and 24 h after treatment, immediately frozen in liquid nitrogen, and stored at –80°C. Each treatment consisted of three biological replicates, with at least 20 plants pooled per group. To measure NO content, 1 g sample was ground in 7 mL of phosphatic buffer solution. The homogenate was then centrifuged at 11,000 g for 10 min, and the supernatant solution was collected. NO content was measured and calculated using the Griess Reagent System kit (Promega Corporation, Madison, WI, USA) with three technical replicates according to the manufacturer’s instructions. Briefly, 50 μL of the supernatant solution and 50 μL of the sulfanilamide solution were mixed, followed by incubation for 10 min at room temperature, protected from light. Then 50 μl of the NED Solution was added, and incubated 10 minutes at room temperature, protected from light. Finally, the absorbance was measured at 540 nm.

### Library construction and sequencing

The materials at 3 h after treatment were used for library construction, with three biological replicates for each treatment, coming to a total of nine libraries. Total RNA was extracted using the EASYspin Plant Total RNA Extraction Reagent (Aidlab, Beijing, China) in accordance with the manufacturer’s instructions and was then stored at −80°C. RNA samples with an A_260_/A_280_ ≥ 1.8, A_260_/A_230_ ≥ 1.8, and RNA integrity number ≥ 6.5 were considered acceptable for library construction. Libraries were constructed using the following steps: 1) Poly (A)-containing mRNA was purified from 6 μg of total RNA using oligo (dT) magnetic adsorption beads. 2) cDNA was synthesized from the bead-bound RNA using oligo (dT) primers. 3) The restriction enzyme *Nla*III was used to digest cDNA, leaving a 4-base overhang (CATG) on the digested cDNA. 4) Unnecessary fragments were washed away, and the Illumina adaptor 1 was ligated to the 5' end of the digested cDNA, which was bead-bounded. 5) The cDNA was digested with *Mme*I, which recognizes the junction between the Illumina adaptor 1 and the *Nla*III site but cuts 17 bp downstream of CATG. 6) Illumina adaptor 2 was ligated to the 3' end of the sequence tag, generating tags with different adaptors at each end. 7) After linear PCR amplification, fragments were purified via polyacrylamide gel electrophoresis. The quantification and qualification of the libraries were detected using an Agilent 2100 Bioanalyzer and an ABI StepOnePlus Real-Time PCR system (Applied Biosystems, USA). The libraries were sequenced using the Illumina HiSeq™ 2000 system. The sequence data of this study have been deposited under SRA accession PRJNA391847.

### Data processing

To obtain clean tags, quality control was performed by filtering out 39-bp adaptor sequences, empty reads, low-quality tags containing unknown sequences (N), tags not equal to 21 bp, and tags with only one copy number. The clean tags were mapped to the predicted coding sequences from the genomic data of *G*. *hirsutum* [27]. Unambiguous clean tags were obtained by filtering out clean tags that mapped to multiple sequences. The number of unambiguous clean tags for each gene was counted and normalized to TPM [28]. All detected genes were annotated based on the non-redundant database of NCBI (ftp://ftp.ncbi.nih.gov/blast/db/FASTA/nr.gz) and the Gene Ontology database (ftp.geneontology.org/pub/go).

To identify DEGs, the following thresholds were used: log_2_ (ratio of two TPM values) ≥ 1, the false discovery rate (FDR) ≤ 0.001, and genes with a tag number > 10 (at least in one library) [29]. Venn diagrams for comparing lists were constructed using an online tool (http://bioinfogp.cnb.csic.es/tools/venny/index.html). Gene Ontology (GO) annotation was performed using the Blast2GO program [30]. GO enrichment was analyzed at the agriGO website (http://bioinfo.cau.edu.cn/agriGO/index.php) via Customized annotation. KEGG Orthology-Based Annotation System (KOBAS 2.0) was then employed to identify the enriched metabolic pathways [31]. To identify plant hormone-related genes, a local BLASTP search was performed with the *Arabidopsis* Hormone Database (AHD2.0, http://ahd.cbi.pku.edu.cn/). To identify TFs, a local BLASTP search was performed with the Database of *Arabidopsis* Transcription Factors (DATF, http://datf.cbi.pku.edu.cn/) [32], and the matched sequences were verified in the Conserved Domain Database of NCBI (CDD, https://www.ncbi.nlm.nih.gov/Structure/cdd/cdd.shtml). The BLAST threshold was set as 1e-5.

### Quantitative real-time polymerase chain reaction (qRT-PCR) analysis

qRT-PCR analysis was performed to verify DGE results. RNA samples used for qRT-PCR were identical to those employed for the DGE experiments. Twenty-five DEGs were selected, and gene-specific primers were designed using Primer Premier 5.0 (S1 Table). *Actin* was used as the internal reference gene. qRT-PCR was carried out using SYBR Green PCR master mix (Roche Applied Science, Germany) on an ABI 7500 real-time PCR system (Applied Biosystems, USA) with three technical replicates. Amplification reactions were initiated with a denaturing step (95°C for 10 min), followed by 40 cycles of denaturing (95°C for 10 s), annealing (60°C for 30 s) and extension (72°C for 40 s).

### Determination of hydrogen peroxide (H_2_O_2_) and malondialdehyde (MDA) contents

Each one g sample was ground in 6 mL of phosphatic buffer solution. The homogenate was then centrifuged at 6,000 g for 25 min, and the supernatant solution was collected. The H_2_O_2_ content was determined via the method of Chen [33]. Then, 600 μL of the supernatant solution was mixed with 2 mL of 0.1% (m/v) titanium chloride in 20% (v/v) H_2_SO_4_. The mixture was next centrifuged at 6,000 g for 15 min, and the supernatant solution was collected. The absorbance was subsequently measured at 410 nm. The MDA content was determined using thiobarbituric acid (TBA) reaction [34]. For this assay, 2.5 mL of the supernatant solution was mixed with 2.5 mL of 0.5% TBA solution. The mixture was then boiled for 15 min and immediately chilled on ice. Then the mixture was centrifuged at 6,000 g for 15 min and the supernatant solution was collected. The absorbance was measured at 532 nm and 600 nm. The MDA content was calculated from the difference in absorbance at 532 and 600 nm using an extinction coefficient of 155 mM^-1^ cm^-1^.

## Results and discussion

### Physiological evaluation of NO donor treatment

**Fig 1:**
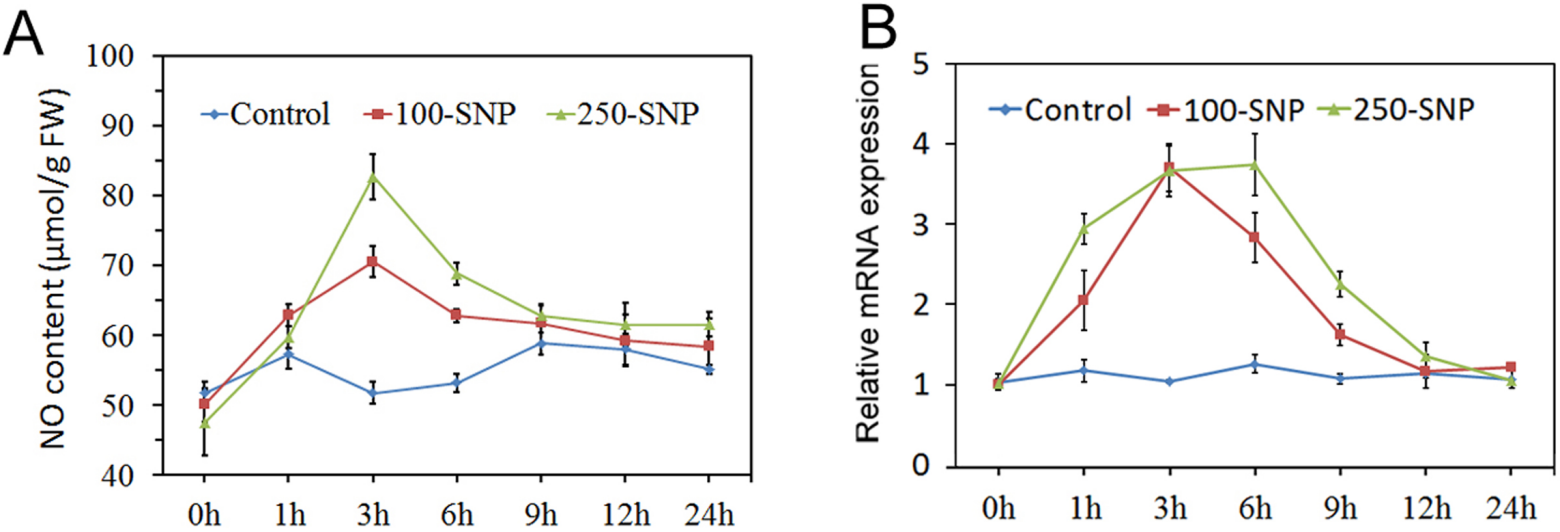
Evaluation of NO donor treatment. **A,** NO contents after treatment with SNP. **B,** Expression pattern of the NO biosynthesis gene *NR* after treatment with SNP.

To ensure the best effect of the treatment, endogenous NO contents were measured according to the time-course after plants were treated with double-distilled water (control), 100 μM SNP, or 250 μM SNP (Fig 1A). Generally, NO content under the 250 μM SNP treatment was higher than that under 100 μM SNP treatment at different times. Compared with control, NO content of both the 100 μM SNP and 250 μM SNP groups first increased, reached a peak at 3 h, then decreased, and remained steady after 9 h. Previous studies have showed that *NR* is the primary gene involved in NO biosynthesis, and can also be induced by SNP treatment. In birch cells, *NR* is up-regulated by SNP treatment [24]. In *Larix olgensis Henry*, two genes annotated to *NR* are also reported to be up-regulated by SNP treatment [25]. These results suggest that exogenous NO can influence endogenous NO synthesis and signals. Therefore, *NR* could be used as a marker gene to evaluate the effect of SNP treatment. Interestingly, a candidate homologous to *NR*, CotAD_40664, was identified in cotton. So we used it to evaluate the effect of SNP treatment at the transcriptional level (Fig 1B). The result showed that *NR* was induced by both 100 μM SNP and 250 μM SNP, especially at 3–6 h after treatment. This result was in accordance with previous studies [24, 25], suggesting that SNP treatment could influence endogenous NO synthesis. Considering the NO content and NR expression level observed after SNP treatment, we performed the next experiment on the plant materials at 3 h after treatment.

### High-throughput tag-sequencing and mapping of DGE libraries

To gain knowledge about the NO-responsive genes, high-throughput tag-sequencing was performed on RNAs from cotton plants treated with ddH_2_O (control), 100 μM SNP (100-SNP), or 250 μM SNP (250-SNP). As a result, 13.4–16.6 million total tags were generated with 0.51–0.58 million distinct tags for each library (S2 Table). After quality control, 13.1–16.3 million total clean tags and 266,789–294,679 distinct clean tags were produced for each library (S2 Table). The distribution of total and distinct clean tag copy numbers showed similar tendencies among the nine libraries (S3 Table). Among the distinct clean tags, ~7% exhibited a copy number more than 100, ~5% exhibited a copy number of 51–100, ~9.5% exhibited a copy number of 21–50, ~10.5% exhibited a copy number of 11–20, ~15% exhibited a copy number of 6–10, and ~53% exhibited a copy number of 2–5. These results are consistent with the rule that most genes are expressed at low levels, whereas minorities of genes are highly expressed under certain conditions. To reveal the molecular events underlying the DGE profiles, the tag sequences of the nine libraries were mapped to the predicted coding sequence based on the genomic data of *G*. *hirsutum* [27]. Nearly 25% of the distinct clean tags (66,342–72,910) could be unambiguously mapped to a gene in the reference gene database (S2 Table). As a result, 41,071–43,218 tag-mapped genes were detected (69.51%–73.14% of the total reference genes), among which 30,439–32,564 (51.52%–55.11% of the total reference genes) genes belonged to unambiguous tag-mapped genes in each library (S2 Table).

We further investigated whether the differences in gene coverage between libraries were due to differences in sample size in terms of unambiguous mapped tags (ranging from 77,541 to 86,903 tags), or to real differences in transcriptome complexity as a function of the number of unique mapped tags (S1 Fig). The analysis clearly showed that, at the depth of sequencing achieved, a plateau was reached for each sample, indicating that the sequencing depth was sufficient for the transcriptome coverage. In addition, Pearson’s rank correlation analysis was performed to evaluate the reproducibility among biological replicates. As shown in S4 Table, the correlations between samples among the biological replicates were good, with values ranging from 0.960 to 0.994. However, the correlations between samples out of the biological replicates were varied, ranging from 0.566 to 0.907. These results illustrate that our data were with high reliability and reproducibility for the biological replicates and the sequencing method.

### Global analysis of the cotton transcriptome in response to NO

**Fig 2:**
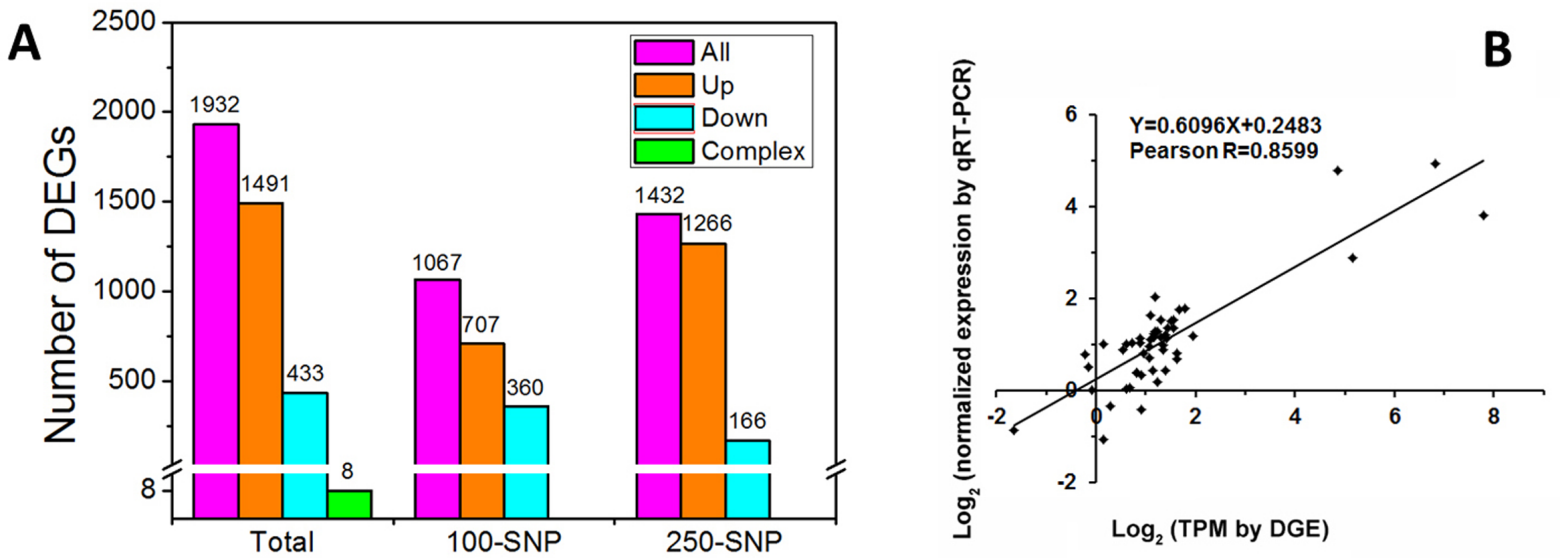
Analysis of the DEGs in response to NO. **A,** Number of DEGs compared with the control. **B,** Comparison of gene expression for 25 DEGs. Data generated using qPCR (*y*-axis) and DGE (*x*-axis) are shown.

Using DESeq package in R programming language, a total of 1,932 DEGs were detected (S5 Table). Among these DEGs, 1,491 were up-regulated, while 433 were down-regulated, and eight showed a complex expression pattern (all eight DEGs were down-regulated by 100 μM SNP and up-regulated by 250 μM SNP). Compared with control, 1,067 and 1,432 DEGs were detected under the 100 μM SNP and 250 μM SNP treatment, respectively (Fig 2A). Among these DEGs, 707 and 1,266 genes were up-regulated, whereas 360 and 166 genes were down-regulated under the 100 μM SNP and 250 μM SNP treatment, respectively. In addition, qRT-PCR was carried out for 25 DEGs to confirm the DGE data. These DEGs included 10 hormone-related DEGs and 15 randomly selected DEGs. The expression patterns obtained via qRT-PCR were consistent with those obtained through DGE analysis, with an overall Pearson R of 0.8599 (ranging from 0.6559 to 0.9999 for each DEG) (P < 0.0001; Fig 2B; S1 Table).

**Fig 3:**
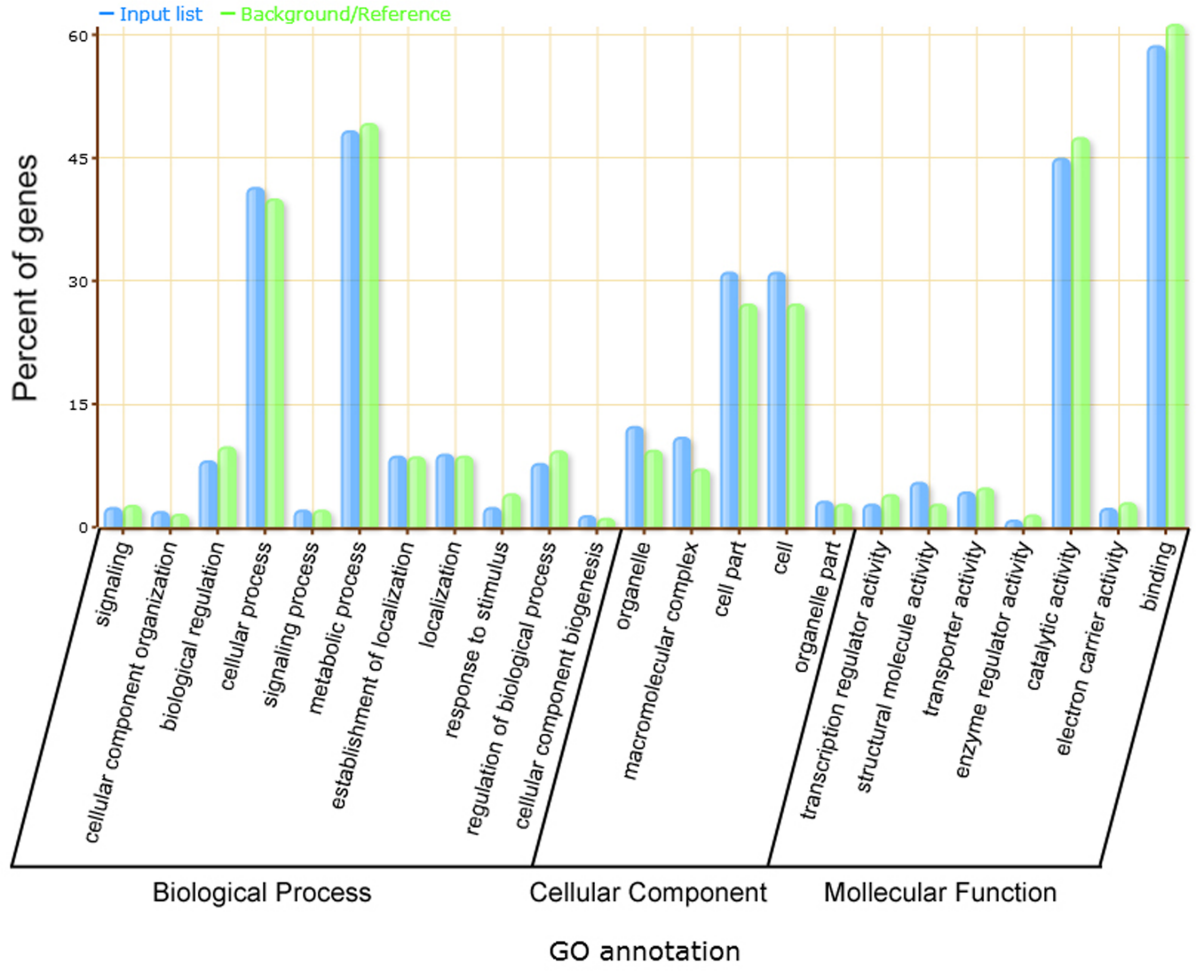
GO enrichment of DEGs in response to NO. The analysis was performed using agriGO.

We used the GO classification system to classify the possible functions of the DEGs. A total of 1,363 (70.5%) DEGs were successfully assigned to at least one GO term associated with biological process, cellular component, or molecular function (S5 Table, S2 Fig). In addition, GO enrichment analysis was performed using agriGO. A total of 14 significantly enriched GO items were identified with an FDR≤0.05, including translation, structural molecule activity, structural constituent of ribosome, ribonucleoprotein complex, ribosome, macromolecular complex, cytoplasm, cytoplasmic part, intracellular part, intracellular non-membrane-bounded organelle, non-membrane-bounded organelle, intracellular, intracellular organelle, and organelle (S6 Table). Among biological process, “metabolic process” (31.47%) and “cellular process” (26.97%) were the two items that exhibited the highest percentage of DEGs. Among cellular component, “cell” (20.29%) and “cell part” (20.29%) were the two items that exhibited the highest percentage of DEGs. Among molecular function, “binding” (38.20%) and “catalytic activity” (29.35%) were the two items that exhibited the highest percentage of DEGs (Fig 3). These results are consistent with previous studies suggesting that binding and catalytic activities operate predominantly at transcriptome level in response to NO [20, 35]. Meanwhile, “transcription regulator activity” also showed a notable percentage of DEGs, demonstrating that TFs might be involved in NO response.

Genes with similar expression patterns are often functionally correlated. To obtain an overview of the expression patterns of the DEGs under different SNP concentrations, we employed MultiExperiment Viewer to cluster the DEGs based on the K-means clustering method. As shown in S3 Fig, these DEGs were clustered into six main clusters. Cluster 1, cluster 2, and cluster 3, which contained 435, 602, and 334 genes, respectively, were up-regulated by both the 100 μM SNP and 250 μM SNP treatment. Cluster 4, which contained 134 genes, was significantly up-regulated by 100 μM SNP, and slightly up-regulated by 250 μM SNP treatment. Cluster 5, which contained 128 genes, was down-regulated by 100 μM SNP, but was not obviously altered by 250 μM SNP treatment. Cluster 6, which contained 299 genes, was down-regulated by both the 100 μM SNP and 250 μM SNP treatment.

In addition, we used KOBAS 2.0 to identify the metabolic pathways in which the NO-responsive genes function. The results are presented in S7 Table and S8 Table. Under the 100 μM SNP treatment, 307 genes were annotated and mapped to 199 KEGG pathways. Among these, the stilbenoid, diarylheptanoid and gingerol biosynthesis, ribosome, starch and sucrose metabolism, and phenylpropanoid biosynthesis pathways were the most enriched pathways (S7 Table). Under the 250 μM SNP treatment, 472 genes were annotated and mapped to 226 KEGG pathways. Among these, plant hormone signal transduction, carbon fixation in photosynthetic organisms, plant-pathogen interaction, carbon metabolism, and ribosome pathways were the most enriched pathways (S8 Table). These results suggested that these pathways might play an important role in the response to NO. Some pathways, such as plant hormone signal transduction and plant-pathogen interaction, have been reported to be involved in NO-responsive processes [7, 13, 36].

### TF family genes are regulated by NO

TFs are key regulators that temporarily and spatially turn on or off the transcription of their target genes through binding certain upstream elements [32]. Previous studies have revealed sets of TF families genes that are regulated by NO [20]. We therefore performed a local BLASTP, using the TF sequences in DATF as database and the translated protein sequences of DEGs as queries. The matched sequences were further confirmed by a Batch CD-search in CDD. As a result, a total of 157 TFs belonging to 36 TF families were identified, among which 85 TFs were differentially expressed under the 100 μM SNP treatment, and 118 TFs were differentially expressed under the 250 μM SNP treatment (S9 Table). The most enriched TF families were bHLH (15), DBP (12), MYB (12), C3H (11), AP2-EREBP (9), NAC (8), WRKY (7), PHD (6), C2C2-CO-like (5), GRAS (5), HSF (5), Orphans (5), and TRAF (5) (Table 1). Furthermore, under the 100 μM SNP treatment, the most enriched TF families were bHLH (7), MYB (6), DBP (4), and C3H (4) among the translated protein sequences of up-regulated DEGs, whereas those the translated protein sequences of down-regulated DEGs were bHLH (3), MYB (3), C3H (2), and C2C2-CO-like (2). Under the 250 μM SNP treatment, the most enriched TF families were bHLH (9), NAC (6), WRKY (6), and MYB (5) among the translated proteins sequences of up-regulated DEGs, and bHLH (2) and MYB (2) among the translated protein sequences of down-regulated DEGs. Orthologs of some identified TF family genes have been reported to be associated with diverse NO-mediated processes of plant growth and development. For example, three of the identified TF-encoding genes (*NAC2*, *NAP*, and *WRKY6)* were shown to be down-regulated during dark-induced leaf senescence in comparison of *nos1/noa1* (an NO-deficient mutant) with wild-type *Arabidopsis* [37]. Meanwhile, three other TF-encoding genes (*AtbHLH38*, *AtbHLH39*, and *AtMYB72)* are strongly induced by Fe deficiency and GSNO treatment (an NO donor) in *A*. *thaliana* [38]. Orthologs of these TF-encoding genes showed marked fold changes after NO treatment, suggesting that NO-responsive genes and pathways might be regulated by these vital TFs.

**Table 1:** Summary of the TFs in response to NO.

TF Family	Total	Up-regulated under 100 μM SNP	Down-regulated under 100 μM SNP	Up-regulated under 250 μM SNP	Down-regulated under 250 μM SNP
**bHLH**	15	7	3	9	2
**DBP**	12	4	0	9	1
**MYB**	12	6	3	5	2
**C3H**	11	4	2	7	1
**AP2-EREBP**	9	3	1	8	0
**NAC**	8	1	1	6	1
**WRKY**	7	1	1	6	0
**PHD**	6	3	1	4	0
**C2C2-CO-like**	5	1	2	3	0
**GRAS**	5	1	0	4	0
**HSF**	5	1	1	4	1
**Orphans**	5	1	1	3	0
**TRAF**	5	2	0	4	0
**AUX/IAA**	4	2	0	4	0
**C2C2-GATA**	4	1	3	1	0
**C2H2**	4	2	1	2	1
**HB**	4	2	1	3	0
**ARF**	3	0	0	3	0
**bZIP**	3	1	1	1	1
**CAMTA**	3	2	0	1	0
**GNAT**	3	1	1	2	0
**SWI/SNF-BAF60b**	3	1	1	2	0
**TCP**	3	2	1	2	1
**ABI3VP1**	2	1	0	1	1
**CCAAT**	2	2	0	2	0
**mTERF**	2	1	0	1	1
**TUB**	2	1	0	2	0
**zf-HD**	2	1	0	1	0
**FAR1**	1	0	0	1	0
**FHA**	1	1	0	1	0
**HMG**	1	1	0	0	0
**LOB**	1	1	0	1	0
**MBF1**	1	0	0	1	0
**MED6**	1	0	0	1	0
**MYB-related**	1	0	1	0	0
**PBF-2-like**	1	1	0	0	0

### Differential expression of hormone-related genes in response to NO

Increasing evidences suggest that NO is an essential component of a wide variety of hormone-regulated processes, including stomatal movement, root growth, senescence, and stress responses [13]. Therefore, we analyzed the NO-responsive DEGs that related to plant hormones. In total, 72 of the DEGs were functionally related to the eight plant hormones, 37 of which were differentially expressed under the 100 μM SNP treatment, while 52 were differentially expressed under the 250 μM SNP treatment (S10 Table). Among these genes, 17, 17, 15, 8, 6, 5, 3, and 1 DEGs were related to the biosynthesis and signal transduction networks of ET, abscisic acid (ABA), auxin (AUX), salicylic acid (SA), brassinosteroid (BR), JA, gibberellin (GA), and cytokinin (CK), respectively (Table 2). Under the treatment of 100 μM SNP, the most abundant hormones were ET (7), ABA (5), AUX (4), and BR (4) among the up-regulated DEGs, whereas they were ABA (5), AUX (2), and BR (2) among the down-regulated DEGs. Under the treatment of 250 μM SNP, the most abundant hormones were AUX (12), ABA (11), and ET (4) in the up-regulated DEGs, whereas they were ABA (1) and AUX (1) among the down-regulated DEGs. These results highlight the role of NO in regulating hormone-related signal transduction pathways.

**Table 2:** Summary of the hormone related genes in response to NO.

Hormone	Total	Up-regulated under 100 μM SNP	Down-regulated under 100 μM SNP	Up-regulated under 250 μM SNP	Down-regulated under 250 μM SNP
**ET**	17	7	1	11	0
**ABA**	17	5	5	11	1
**AUX**	15	4	2	12	1
**SA**	8	2	1	5	0
**BR**	6	4	2	4	0
**JA**	5	2	1	4	0
**GA**	3	0	0	3	0
**CTK**	1	0	1	0	0

**Fig 4:**
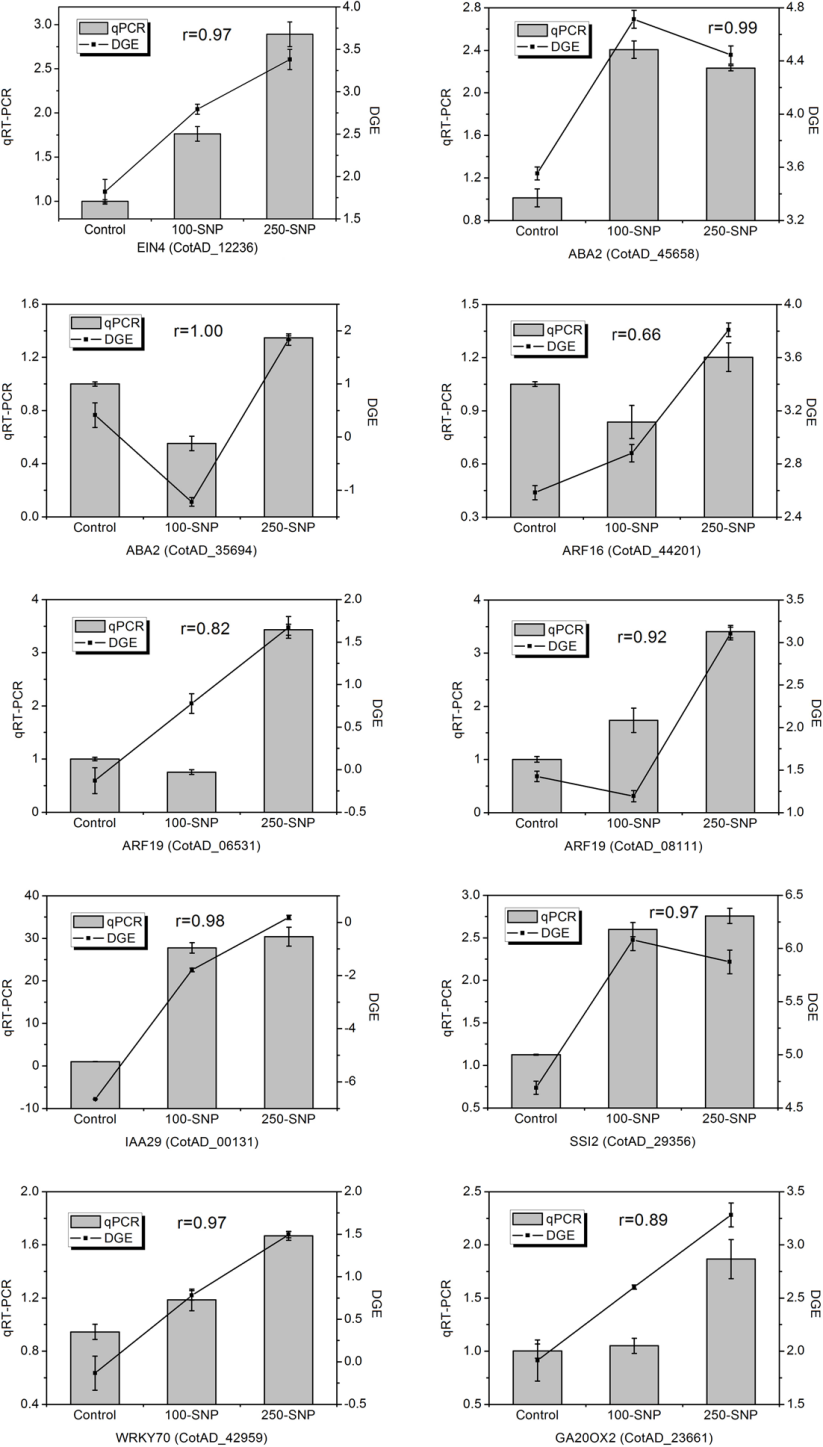
Expression levels of candidate DEGs related to hormone biosynthesis and signaling pathways.

It is reported that NO induces the expression of genes involved in ET biosynthesis, thus regulating the ET signal pathway [36, 39]. In *A*. *thaliana*, genes of *ET-insensitive protein* (*EIN*) are pivotal in ET signal transduction. We found that an ortholog of *EIN4*, CotAD_12236, was induced by both the 100 μM SNP and 250 μM SNP treatments (Fig 4). NO is required in ABA signaling to induce stomatal closure and inhibite light-induced stomatal opening [40]. We identified two orthologs of the ABA biosynthesis gene *ABA2* among our results: CotAD_35694 and CotAD_45658. CotAD_35694 was down-regulated by 100 μM SNP, but up-regulated by 250 μM SNP, whereas CotAD_45658 was up-regulated by both 100 μM SNP and 250 μM SNP. It has also been demonstrated that NO is involved in mediating iron deficiency-induced root morphology changes together with AUX in *A*. *thaliana* [15]. We also identified four NO-responsive DEGs associated with the AUX signal pathway. Three of them were orthologous to auxin response factors (CotAD_44201, CotAD_06531, CotAD_08111), while one was orthologous to indole-3-acetic acid inducible 29 (CotAD_00131). All four of these DEGs were up-regulated by 100 μM SNP or 250 μM SNP. NO functions together with JA and SA in modulating plant defense responses, including the responses to pathogens and abiotic stresses (e.g., low temperature, drought, and high salinity) [41]. One DEG related to JA biosynthesis (CotAD_29356, orthologous to *SSI2*) and one DEG related to JA signal transduction (CotAD_42959 orthologous to *WRKY70*), were identified. Both were up-regulated by 100 μM SNP and 250 μM SNP. Although we did not find obvious evidence of an interaction of NO with GA, a key DEG in GA biosynthesis, CotAD_23661, which is orthologous to *GA20OX2*, was also identified, with an up-regulation by 250 μM SNP. The expression of these hormone-related DEGs was verified through qRT-PCR (Fig 4), where results for nine of the ten DEGs were consistent with the DGE data, with a Pearson correlation value above 0.8. All the above findings clearly indicate that NO regulates hormone-related signal transduction pathways to influence plant biological processes.

### Stress-responsive genes are regulated by NO

**Fig 5:**
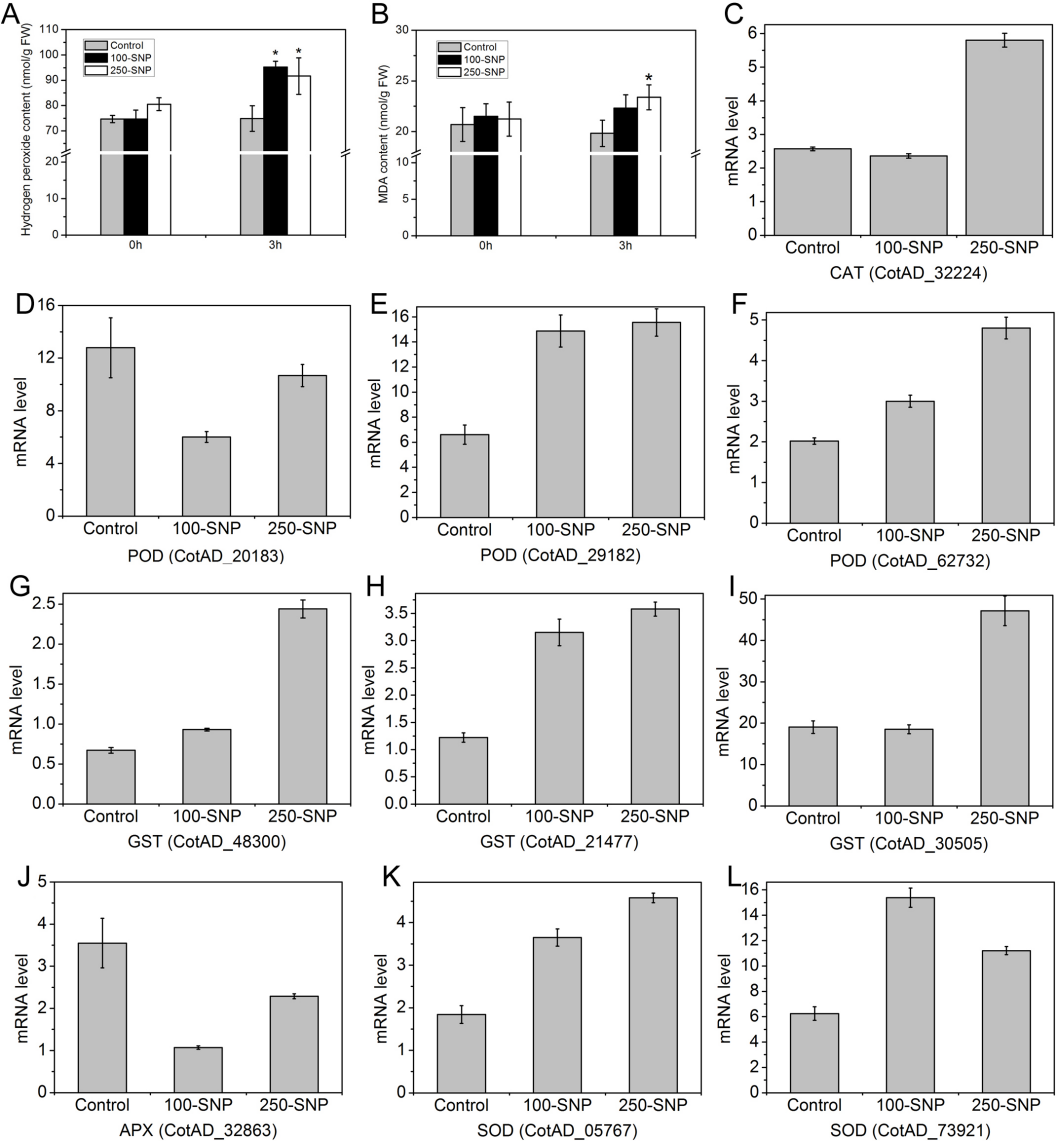
NO is involved in the stress response. **A,** Changes in hydrogen peroxide content after treatment with SNP. **B,** Changes in malondialdehyde content after treatment with SNP. **C-L,** Expression level of DEGs related to stress responses and defense process.

Based on the identification of TFs regulated by NO, we found several TF families were involved in biotic and abiotic stress responses, such as NAC [42], WRKY [43], MYB [44], and HSF [45]. At the same time, ABA, SA, JA and ET are major hormones known to play major roles in mediating the plant defense response against pathogens and abiotic stresses [46]. The identification of hormone-related DEGs provides additional evidence that NO might play a role in the regulation of plant stresses. We therefore determined the contents of H_2_O_2_ and MDA, two widely employed physiological markers that are induced by various stresses [47], after treatment with SNP. Consistently, the H_2_O_2_ content showed significant increases after 3 h treatment with 100 μM SNP and 250 μM SNP compared with the control (P<0.05) (Fig 5A). The MDA content was also increased after 3 h 100 μM SNP treatment, and showed a significant increase after 3 h 250 μM SNP treatment (Fig 5B). At the mRNA level, a gene encoding catalase (producing H_2_O_2_) (CotAD_32224) was found to be up-regulated by 250 μM SNP (Fig 5C). In addition, nine genes encoding antioxidant defense proteins showed altered regulation after treatment with SNP (Fig 5D–5L). These proteins included peroxidase (CotAD_20183, CotAD_29182, CotAD_62732), glutathione S-transferase (CotAD_48300, CotAD_21477, CotAD_30505), ascorbate peroxidase (CotAD_32863), and superoxide dismutase (CotAD_05767, CotAD_73921). With the exception of CotAD_20183 and CotAD_32863, the remaining seven genes showed up-regulation under the 100 μM SNP or 250 μM SNP treatment. In a previous study, hundreds of DEGs, including *CAT* and *APX*, were found to be involved in plant defense against various biotic and abiotic stresses after treatment of *Arabidopsis* leaves with S-nitrosocysteine (CysNO, another NO donor) [20]. In tobacco, significant cross-talk between NO and H_2_O_2_ was found, and target genes of the combined action of NO and H_2_O_2_ during the induction of plant cell death were identified [21]. In birch, the H_2_O_2_ content was also increased after SNP treatment, with genes encoding FeSOD, MnSOD, and CZSOD up-regulated after SNP treatment compared with the control [24]. Our results were consistent with these studies, suggesting the involvement of NO in plant stress responses and defense.

### Comparison of the NO-responsive genes of cotton with other plants

The identification of NO-responsive genes at a large-scale transcriptome level has been performed in several plant species thus far, including *A*. *thaliana *[17–20, 47], *N*. *tabacum* [21], *M*. *truncatula* [22], *L*. *chinensis* [23], *B*. *platyphylla* Suk.[24], and *L*. *olgensis Henry* [25]. We compared the experimental design and results of these 10 studies with those of our study, including examination of the treatment method and time, species, sampled tissues, sequencing method, number of total genes, and number of DEGs. As shown in Table 3, the treatment methods were divergent between these studies. In eight studies, the authors chose SNP as the NO donor to perform the experiment. Among other NO donors chosen for the experiments, GSNO, CysNO, and NOR3 were used twice, once, and once, respectively. Besides, gaseous NO was used to perform the experiment in one case. All the NO donors as well as gaseous NO are acceptable, however, their responsive genes exhibit differences. For example, in the Ferrarini et al. study, among genes responsive to either GSNO or SNP, only approximately 11% were common to both groups in the leaves and 1.6% in the roots. Thus, it appears that the modulation of gene expression is dependent on the form of NO produced (NO^+^ released by SNP or NO^−^released by GSNO) [22, 48]. In addition, even when the same NO donor was used, the treatment times, species, and sampled tissues differed among these studies. This may due to the specific purposes of the studies, and the most effective treatment times in different species. The number of total genes and DEGs identified is increasing with the development of new sequencing technologies. For example, using the cDNA–AFLP, hundreds of DEGs have been identified [17, 21]; whereas using the RNA-Seq, thousands of DEGs have been identified [24, 25].

**Table 3:** Overview of the transcriptome analysis of NO responsive genes in plants.

Author-year	Treatment method and time	Species	Sample Tissue	Sequence method	Number of Total genes	Number of DEGs	With quality control data
**Polverari et al. 2003**	1 mM SNP; 10 min, 1 h, and 3 h	*Arabidopsis*	leaves	cDNA -AFLP	2,500 cDNAs	120	-
**Parani et al. 2004**	0.1 mM and 1.0 mM SNP; 3 h	*Arabidopsis*	leaves	microarray	Not mentioned	Up: 342; down: 80	-
**Zago et al. 2006**	1 mM SNP; 0 h, 1 h, and 3 h	*Nicotiana tabacum*	leaves	cDNA-AFLP	8,000	214	-
**Palmieri 2007**	1250 ppm gaseous NO; 10 min 0.5 mM NOR3; 1 h and 3 h	*Arabidopsis*	Seedlings Cell suspension cultures	microarray	Not mentioned	254	-
**Ferrarini et al. 2008**	1 mM SNP or 0.5 mM GSNO; 3 h	*Medicago truncatula*	Roots and leaves	MtNO array	999	497	-
**Begaramorales et al. 2014**	1 mM GSNO; 3 h	*Arabidopsis*	Roots and leaves	RNA-Seq	Not mentioned	3,263	Yes
**Liu et al. 2014**	0.5 mM SNP; 10 h	*Litchi chinensis*	Floral buds	SSH cDNA library	728 EST clones	25 clones	-
**Zeng et al. 2014**	1 mM SNP; 12 h	*Betula platyphylla*	Cell suspension Culture	RNA-Seq	34,100 unigens	1,374	Yes
**Hu et al. 2015**	167.8 μM SNP; 5 h	*Larix olgensis*	seedlings	RNA-Seq	58,080 unigenes	Up: 2,671; down: 3,674	Yes
**Hussain et al. 2016**	1 mM CysNO; 6 h	*Arabidopsis*	leaves	RNA-Seq	32,535 genes	6,436 genes	Yes
**This study**	100 and 250 μM SNP; 3 h	cotton	cotyledons	Tag-sequencing	21,652 genes	1,932 genes	Yes

As the treatment methods and sampled tissues used greatly influence gene expression results, we made a comparison of the 100 μM SNP-responsive genes in our study to the 100 μM SNP-responsive genes reported in *A*. *thaliana* [18]. Both experiments were irrigated with 100 μM SNP, and the overground tissues were harvested 3 h after treatment. Among the up-regulated genes, we identified 15 genes (out of 707 DEGs) from our data that aligned with 13 genes (out of 123 DEGs) from the *A*. *thaliana* data (Table 4). These aligned genes included some well-defined genes induced by NO. For example, the gene encoding ferritin (CotAD_54212, orthologous to AT5G01600) was induced, confirming previous reports that NO regulates iron homeostasis through *Arabidopsis ferritin 1* [11, 12, 14]. An ethylene-responsive TF, *ERF13* (CotAD_74946, orthologous to AT2G44840), belonging to the AP2/EREBP TF family, was also identified. TFs in this family participate in the regulation of disease resistance [49], suggesting that NO may regulate disease resistance pathways through interaction with *ERF13*. In addition, two genes related to calcium signaling were identified (CotAD_56107, orthologous to AT1G76650, and CotAD_28267, orthologous to AT2G41010). One report indicated that NO functions in signaling and acts upstream of *AtCaM3* in heat tolerance in *A*. *thaliana* [16], providing evidence supporting our findings that genes in the calcium signaling pathway can be induced by NO. Among the down-regulated genes, we identified only two genes (out of 360 DEGs) from our data that aligned with two genes (out of 50 DEGs) from the *Arabidopsis* data (Table 4). However, there is little genetic or physiological evidence that these two genes could be induced by NO.

**Table 4:** List of genes differential expressed both in cotton and in *Arabidopsis* in response to 100 μM SNP.

ID	Orthologs in *Arabidopsis*	evalue	Expression pattern	Annotation
**CotAD_54212**	AT5G01600	2E-107	Up	ATFER1, FER1, ferretin 1
**CotAD_74946**	AT2G44840	9E-31	Up	ATERF13, EREBP, ERF13, ethylene-responsive element binding factor 13
**CotAD_56107**	AT1G76650	2E-31	Up	CML38, calmodulin-like 38
**CotAD_28267**	AT2G41010	2E-36	Up	ATCAMBP25, CAMBP25, calmodulin (CAM)-binding protein of 25 kDa
**CotAD_12787**	AT1G17100	8E-41	Up	SOUL heme-binding family protein
**CotAD_25972**	AT1G22190	4E-37	Up	Integrase-type DNA-binding superfamily protein
**CotAD_66141**	AT1G60190	0	Up	ARM repeat superfamily protein
**CotAD_68155**	AT1G64780	0	Up	ATAMT1;2, AMT1;2, ammonium transporter 1;2
**CotAD_67449**	AT2G21650	8E-26	Up	MEE3, ATRL2, RSM1, Homeodomain-like superfamily protein
**CotAD_10747**	AT2G24540	2E-09	Up	AFR, Galactose oxidase/kelch repeat superfamily protein
**CotAD_72617**	AT2G24540	2E-83	Up	AFR, Galactose oxidase/kelch repeat superfamily protein
**CotAD_08677**	AT2G47180	0	Up	AtGolS1, GolS1, galactinol synthase 1
**CotAD_47656**	AT3G28340	7E-172	Up	GATL10, galacturonosyltransferase-like 10
**CotAD_17526**	AT4G15210	8E-151	Up	ATBETA-AMY, AT-BETA-AMY, RAM1, BMY1, BAM5, beta-amylase 5
**CotAD_36104**	AT4G15210	5E-149	Up	ATBETA-AMY, AT-BETA-AMY, RAM1, BMY1, BAM5, beta-amylase 5
**CotAD_29042**	AT1G15670	3.00E-125	Down	Galactose oxidase/kelch repeat superfamily protein
**CotAD_53846**	AT5G25350	3.00E-14	Down	EBF2, EIN3-binding F box protein 2

## Conclusion

In conclusion, we described transcriptional changes in response to NO in upland cotton, using high-throughput tag-sequencing. A total of 1,932 DEGs were identified, among which 1,067 and 1,432 were regulated by 100 μM SNP and 250 μM SNP, respectively. qRT-PCR on 25 DEGs was performed to validate the data and consistent results were obtained. GO classification and KEGG pathway were analyzed to obtain additional knowledge about the transcriptome data. Furthermore, we identified 157 DEGs belonging to 36 TF families and 72 DEGs related to eight hormones. Several TF families (NAC, WRKY, MYB, and HSF) and hormones (ABA, SA, JA, and ET) are involved in stress responses. In addition, H_2_O_2_ and MDA contents were shown to increase, and related genes showed marked up-regulation after treatment with SNP. These results suggest that NO might play a role in plant stress response. At last, comparison of NO-responsive genes recorded in the present study with the results of previous studies led to the identification of dozens of vital DEGs that shared between *A*. *thaliana* and cotton. To our knowledge, this is the first study that performs deep-sequencing analysis of the response to NO in cotton. These data provide a foundation for future studies concerning NO and stress response in cotton. In addition, some DEGs identified in this study represent good candidates for further functional analysis in cotton.

## Supporting information

S1 Fig Saturation analyses of the DGE libraries of control (A-C), 100 μM SNP (D-F), and 250 μM SNP (G-I) treatment.
(TIF)

S2 Fig Biological processes (A), cellular component (B), and molecular function (C) of NO-responsive genes involved according to GO annotation.
(TIF)

S3 Fig K-means clusters of the NO-responsive genes. (TIF)

S1 Table Validation of DGE data through qRT-PCR analyses. (XLSX)

S2 Table Tag mapping and normalization of tag counts. (XLSX)

S3 Table Distribution of total and distinct clean tags in the DGE libraries. (XLSX)

S4 Table Pearson’s rank correlation of nine libraries. (XLSX)

S5 Table TPM value and annotation of DEGs in response to NO. (XLSX)

S6 Table GO enrichment analysis using agriGO. (XLSX)

S7 Table KEGG pathways enriched under the 100 μM SNP treatment. (XLSX)

S8 Table KEGG pathways enriched under the 250 μM SNP treatment. (XLSX)

S9 Table List of transcription factors regulated by NO. (XLSX)

S10 Table Annotation of hormone-related DEGs. (XLSX)
