# Supplementary figures and images for: Transcriptome analysis of nitric oxide-responsive genes in upland cotton (Gossypium hirsutum)
Source: PLoS One. 2018 Mar 7;13(3):e0192367. doi: 10.1371/journal.pone.0192367 (PMC5841646; doi:10.1371/journal.pone.0192367)

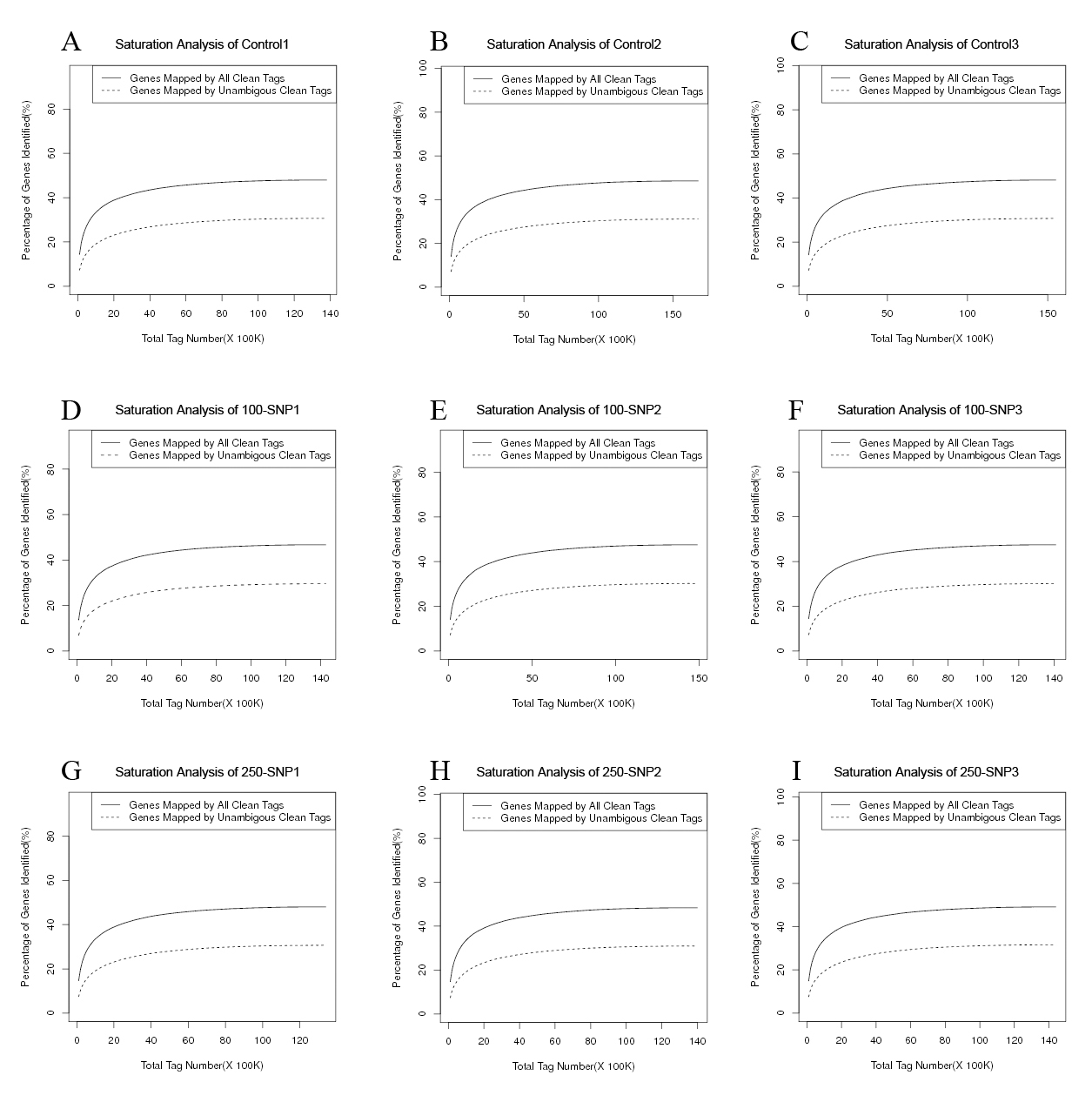

Supplement: S1 Fig — Saturation analyses of the DGE libraries of control (A-C), 100 μM SNP (D-F), and 250 μM SNP (G-I) treatment. (TIF) [file pone.0192367.s001.tif]

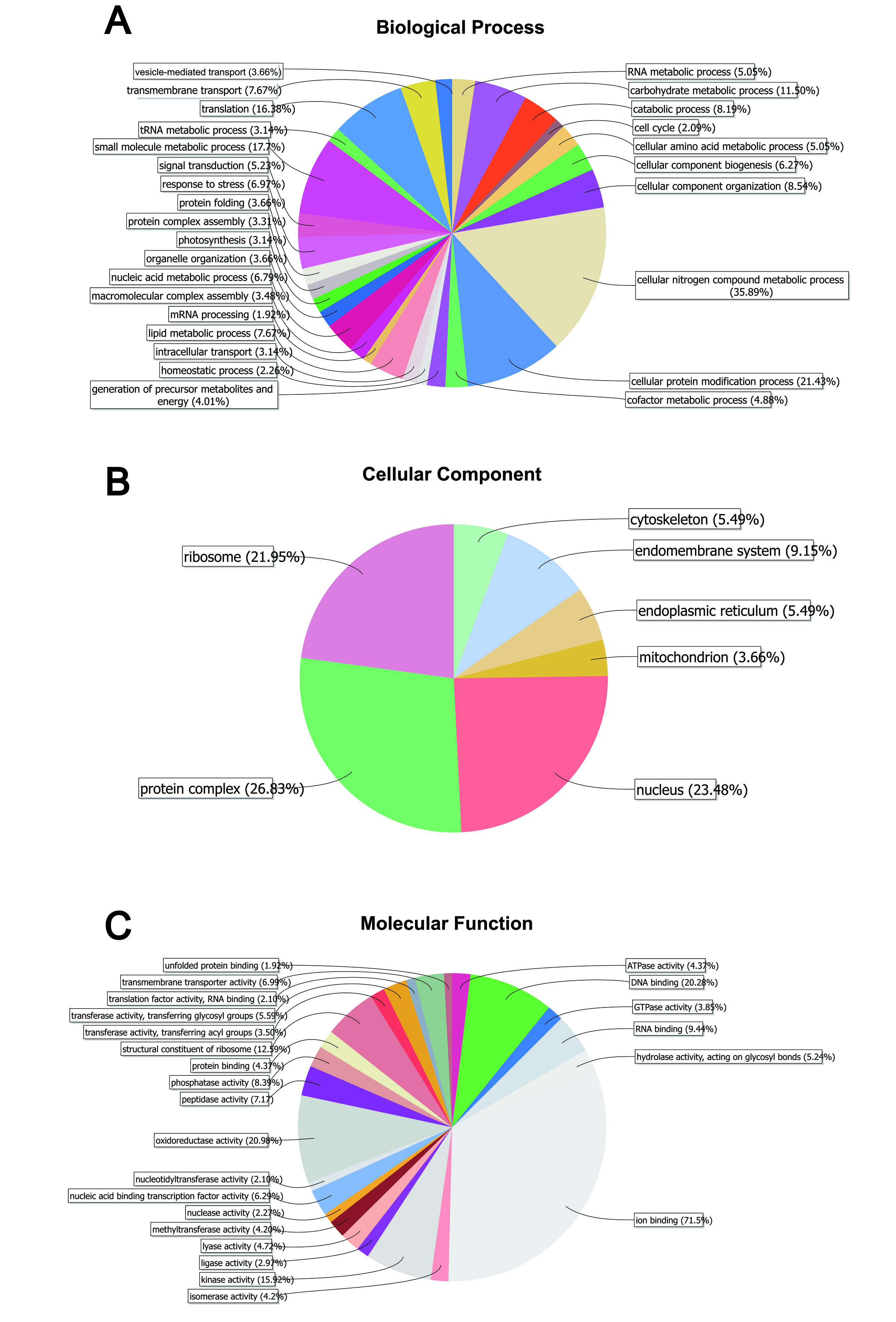

Supplement: S2 Fig — Biological processes (A), cellular component (B), and molecular function (C) of NO-responsive genes involved according to GO annotation. (TIF) [file pone.0192367.s002.tif]

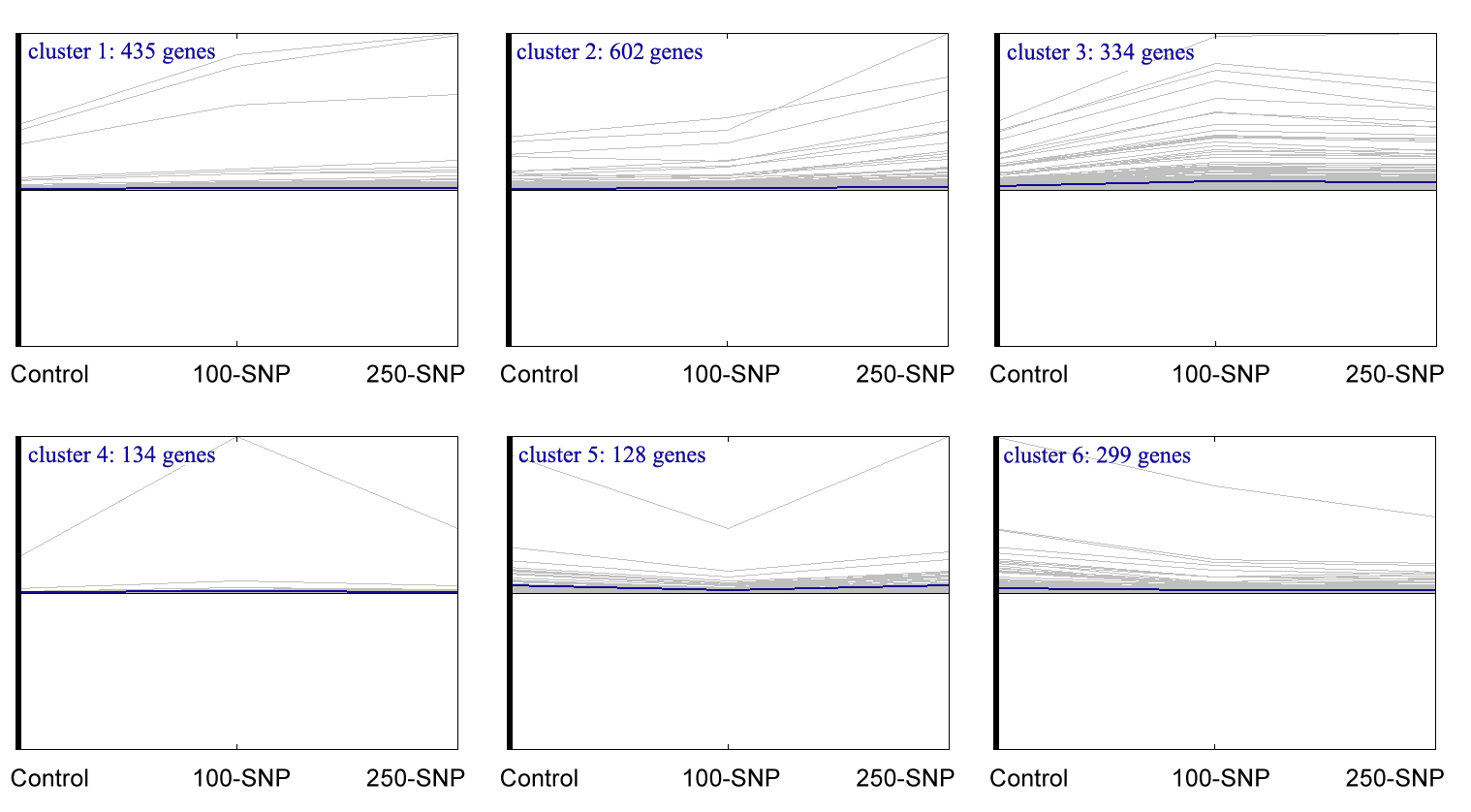

Supplement: S3 Fig — (TIF) [file pone.0192367.s003.tif]
